# Early history of the *European Journal of Neurology*. A mirror of the evolution of Europe after 1989

**DOI:** 10.1111/ene.16236

**Published:** 2024-02-20

**Authors:** François Boller, Anne Petrov, Per Soelberg Sørensen

**Affiliations:** ^1^ Department of Neurology George Washington University Medical School Washington DC USA; ^2^ Silver Spring Maryland USA; ^3^ Department of Neurology Rigshospitalet, University of Copenhagen Copenhagen Denmark; ^4^ Department of Neurology, Danish Multiple Sclerosis Center Rigshospitalet, Copenhagen University Hospital Copenhagen Denmark

**Keywords:** European Academy of Neurology, European Federation of Neurological Societies, European Neurological Society, European neurology, history

## Abstract

This paper retraces the early history of the *European Journal of Neurology* (EJN), as it is about to enter its 30th year. It describes how our discipline organized itself during the latter part of the 20th century in Europe. In some ways, the creation and the evolution of the journal parallel the process of unification of Europe in its current form in the late 1980s and early 1990s. It started as a new journal with no impact factor and no indexation. It grew progressively thanks to the support of the European Federation of Neurological Societies (EFNS) and from the European scientific community The progressive merging of EFNS with the European Society of Neurology and the creation of the European Academy of Neurology were essential for reaching the current prominence of EJN within neurological publishing and for making it the widely heard official voice of European neurology.

The *European Journal of Neurology* (EJN), the official Journal of the European Academy of Neurology, is about to enter its 30th year. It seems fitting to retrace its early history, also because it reflects how our discipline organized itself during the latter part of the 20th century. In some ways, the creation and the evolution of the journal parallel the process of unification of Europe in its current form in the late 1980s and early 1990s. As pointed out in a recent review [1], the time was ripe for creating a society that would unify and coordinate the activities of the neurological societies of each European country. This led to the creation of the European Federation of Neurological Societies (EFNS) in 1991, a realization due to the efforts of many neurologists, first and foremost Professor Franz Gerstenbrand (1924–2017). He was proud to say that he was originally from Moravia (now Czech Republic), a region that has given birth to such characters as Sigmund Freud and Milan Kundera. He completed his medical training in only 4 years under difficult post‐war circumstances in a city, Vienna, which was still feeling the sequels of the tension between the West and the Soviet Union so well depicted in Graham Greene's novel, *The Third Man*, and Orson Wells' film inspired by the novel. His specialty training was at the Psychiatric‐Neurological University Department of Vienna University Hospital (Psychiatrisch‐Neurologische Universitäts‐Klinik Wien), chaired by Professor Hans Hoff. In 1976, he was appointed Chair of the University Clinic for Neurology in Innsbruck. He was a man of vision with a number of skills. In addition to being an excellent neurologist, he contributed to the fields of history of neurology, medical ethics and in his later years aerospace medicine. The creation of the new Society of which he was the first President was a tribute to his outstanding ability as an organizer. Just to think that neurologists with completely different backgrounds and history, from Kiev and Warsaw to Lisbon, from Kuopio (Finland) to Paris, Lausanne and beyond, could work together required audacity. One important goal of the Society was to have its own journal rather than just collaborating with an existing journal. This initiative was entrusted to Jes Olesen, Professor of Neurology at the University of Copenhagen and a world renowned expert in the field of headaches. In early 1993, contact was established with several international publishers and a positive response came from Rapid Communication of Oxford (RCO), a very dynamic publisher known for its catalogue of neurology books and for the journal *NeuroReport*. Contact was established and collaboration started with its energetic representatives, Anthony Gresford, Lucy Gill and later, when the administrative office of RCO moved to London, Caroline Black. A contract was signed giving RCO the right to publish the journal on behalf of the EFNS for 5 years with full ownership of the journal by EFNS. At the end of the 5‐year period, in 2000, publishing was entrusted to Blackwell Science Ltd which in turn merged and became Wiley‐Blackwell, the current publisher of EJN, in 2007. Gavin Sharrock was instrumental in ensuring a smooth transition during those years.

Editorship of the new journal was entrusted to two well‐established neurologists. François Boller MD PhD was Director of an INSERM Unit in Paris dedicated to the neurology and neuropsychology of cerebral aging. Some members of the Unit had been part of a group created by one of the founders of modern neuropsychology, Henry Hécaen, in 1970. François Boller had trained in Italy and in the USA and had edited a well‐known textbook series, the *Handbook of Neuropsychology*. The other Editor of EJN was Per Soelberg Sørensen MD PhD, Professor of Neurology at the University of Copenhagen and senior consultant at the Department of Neurology, Rigshospitalet, Copenhagen. In the early 1990s, he had founded the Danish Multiple Sclerosis Center at the Rigshospitalet, with a large multidisciplinary clinic, offering highly specialized therapies for multiple sclerosis. A meeting hosted by Professor Olesen involving Anthony Gresford and the two future Editors took place in Copenhagen in the Fall of 1993, after which things proceeded very quickly. As for the language of the journal, it was decided to use English exclusively, even though at the time several journals were still multilingual. We chose the cover (Figure 1) and the format. An early task was to assemble the Editorial Board. The choice was based first and foremost on the scientific level and reliability of the members, but geography was also taken into account so as to be representative of the growing geopolitical body of Europe. The Board included 40 prominent neurologists from various countries including some outside of Europe: USA and Canada, Argentina, Tunisia and Israel. We also had a group of prominent Associate Editors. We wrote the scope of the journal (this has remained substantially unchanged) as well as instructions for authors to fit the journal format (Harvard: name/date style). There was also a calendar of events.

**FIGURE 1:**
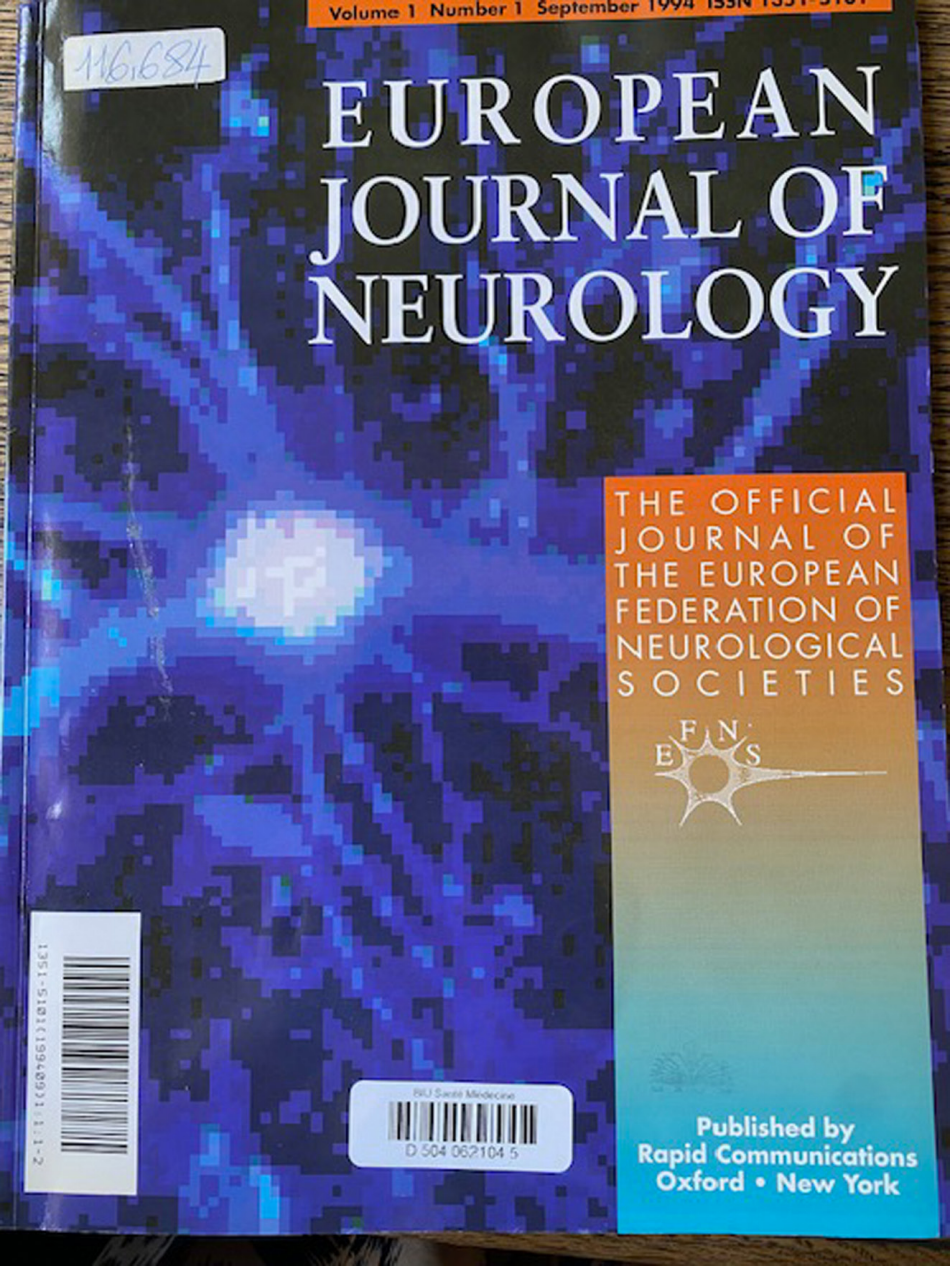
Cover of EJN first issue, September 1994.

Two editorial offices were created. The Paris office was managed by Anne Petrov who had recently left a fledgling career in research nuclear physics in order to write a dissertation on institutionalized science, at the Sorbonne in Paris. The Copenhagen office was managed by Liselotte Albertsen, who apart from having been personal assistant for Per Soelberg Sørensen for several years had training as trilingual correspondent and, previous to that, as a flight attendant. Anne and Liselotte worked splendidly together and proved to have an outstanding ability to communicate with authors submitting manuscripts and with reviewers. They both contributed significantly to the challenge of developing and eventually fully automating the submission process, as well as shortening the turnaround time between submission and publication. Thanks to the linguistic skills of the two offices, we were able to provide scientists with considerable in‐house English editing. Of course authors were sometimes dismayed by our editorial decisions. Few people like to have their papers rejected. On one occasion, after a teaching course given by the two Editors at an EFNS meeting, a lady from the audience approached the speakers at the end of the course. She said she had come with the intent of throwing rotten tomatoes because she was extremely unhappy to have received a rejection. However, she had found us ‘very nice’ and had refrained from her public demonstration. On the other hand, many authors thanked us for both fast turnaround and linguistic help.

This was ‘only’ 30 years ago, yet the world, particularly the publishing world, was quite different from what it is now. Internet did exist but it was not nearly as universal as today and the Worldwide Web (www) was in its infancy. The use of fax was prevalent, but on the whole rather cumbersome, slow and not always reliable. As for personal computers, they were often quite complex to use. The Paris office was proud of its new Macintosh which now would be seen as ridiculously obsolete and slow, good for being in a museum. Very few people had an email address and cell phones were rare. At that time, submissions were still via the Post Office ‘snail mail’. The very first submission to the EJN came from a British paediatric neuroophthalmologist. It was not clear how she had heard about us…

The original EJN instructions asked authors to submit three copies of manuscripts ‘in machine readable form’. Almost as an afterthought, the instructions added at the end ‘We will accept submission of either IBM or Apple Macintosh compatible disks.’ At the time, the two systems were different enough that authors were requested to specify which word‐processing system they had used. It was only later that floppy disks became widely used, replaced by rewritable CDs after 2000. A few years later, electronic submission became mandatory for EJN, as it is for most scientific journals. Obviously, there has been a great deal of progress in terms of speed, getting rid of galley proof reading and other hurdles. The current system is less personalized and requires some perseverance. Fortunately, EJN has a personalized assistance office which rescues naïve submitters and reviewers from difficulties with registration and from the nightmare of ‘forgotten’ passwords.

The first issue of EJN was published in September 1994, a record time since it was less than a year after the initial ‘founding’ meeting. Of course, the most difficult task was to obtain quality papers. The two Editors encouraged well‐established authors and research groups to submit to EJN their high‐calibre original research papers. As a new journal, EJN had no impact factor and was not indexed in PubMed, MEDLINE or any other database. We could only promise potential authors that inclusion in PubMed and assignment of an impact factor would be done retroactively within a few years. Highly esteemed researchers were encouraged to write review articles on some hot topic. For our first review article, we persuaded the renowned multiple sclerosis researcher Tomas Olsson to write a review on ‘Role of cytokines in multiple sclerosis and experimental autoimmune encephalomyelitis’, now a classic in the field. Several well‐known neurologists in the areas of migraine, dementia, AIDS, stroke and multiple sclerosis contributed original research articles to make the inaugural and subsequent issues of EJN impressive and successful.

In subsequent years, the editorial offices received an increasing number of articles. Precise statistics concerning the geographical origin and topics of the submissions are not available. It was noticeable that several papers came from countries that had joined or were in the process of joining the European Union, particularly the Czech Republic and Slovakia (Czechoslovakia had split just as EJN was borne), Poland and Hungary. The published papers covered all the topics included in the scope of the journal as well as the history of neurology and, increasingly, guidelines. These were particularly important because most importantly they would be frequently quoted in the future. Case reports constituted a large proportion of the submissions, but as a rule they were accepted only if they offered some new insight into the field. Within less than 2 years, the EJN was indexed in PubMed and, in 1997, EJN had its first impact factor: 0.641. Six short years later, in 2003, the impact factor had reached 2.00, thereafter slowly but surely climbing to its current level: 6.288.

Anne Petrov stepped down in 2003 to develop windfarms in southwest France. Soon thereafter, in late 2003, Dr Sørensen and the Copenhagen office manager also stepped down and were replaced by Professor Matti Hillbom from Oulu University, a relatively new Institution but now one of the largest universities of Finland. Professor Hillbom is a well‐known specialist in alcohol and strokes. Office management was entrusted to Sirkka‐Liisa Leinonen. It was quite an event to have an Editorial Board meeting in that far north part of the world. In 2005 François Boller left for a new career in the USA. Professor Anthony Schapira MD DSc, Head of the Department of Clinical and Movement Neurosciences, Professor of Neurology at University College London, became Editor‐in‐Chief. In turn, in 2022 he was replaced by the current Editor‐in‐Chief, Professor Didier Leys MD PhD, Professor of Neurology at the University of Lille. He is a world‐renowned clinician/researcher in the field of strokes. He had been the first general secretary of the European Academy of Neurology and a former president of the French Society of Neurology.

We received essential help from many sources. The Paris office was housed within an INSERM Research Unit located at a major Paris hospital (Centre Hospitalier Sainte‐Anne). The Copenhagen office was located within the Department of Neurology at the University of Copenhagen and an agreement was made that secured the editorial office access to many facilities.

Much of the journal development and activities were related to those of EFNS. In this context we want to recognize the contribution of the EFNS Executive Directors, Elizabeth (‘Lisa’) Mueller and Anja Sander. Dr Friederike (‘Uschi’) Tschabitscher had been the very soul of the Society since its beginnings and up to the time of her untimely death in 2003. A Prize for young neurologists commemorates her extraordinary work.

Each EFNS meeting was an occasion for the two Editors to ‘advertise’ and to urge our colleagues to send articles to EJN. Throughout these years, there had been increasing cooperation with many members of the ‘sister’ society, the European Neurological Society. The progressive merging of the two Societies and the creation of the European Academy of Neurology were essential for reaching the current prominence of EJN within neurological publishing and for making it the widely heard official voice of European neurology.

So, from these beginnings, it seems we have all succeeded. Happy 30th to EJN!

## AUTHOR CONTRIBUTIONS

**François Boller:** Conceptualization. **Anne Petrov:** Methodology. **Per Soelberg Sørensen:** Investigation.

## CONFLICT OF INTEREST STATEMENT

None.

## Data Availability

Research data are not shared.
